# Impact of the COVID-19 pandemic on the coverage and timeliness of routine childhood vaccinations in the Gambia, 2015–2021

**DOI:** 10.1136/bmjgh-2023-014225

**Published:** 2023-12-26

**Authors:** Oghenebrume Wariri, Chigozie Edson Utazi, Uduak Okomo, Alieu Sowe, Malick Sogur, Sidat Fofanna, Esu Ezeani, Lamin Saidy, Golam Sarwar, Bai-Lamin Dondeh, Kris A Murray, Chris Grundy, Beate Kampmann

**Affiliations:** 1 Vaccines and Immunity Theme, MRC Unit The Gambia at the London School of Hygiene and Tropical Medicine, Banjul, The Gambia; 2 Department of Infectious Disease Epidemiology, London School of Hygiene and Tropical Medicine, London, UK; 3 Vaccine Centre, London School of Hygiene and Tropical Medicine, London, UK; 4 WorldPop, School of Geography and Environmental Science, University of Southampton, Southampton, UK; 5 Southampton Statistical Sciences Research Institute, , University of Southampton, Southampton, UK; 6 MARCH Centre, London School of Hygiene and Tropical Medicine, London, UK; 7 Expanded Programme on Immunization, Ministry of Health and Social Welfare, Banjul, The Gambia; 8 Health and Demographic Surveillance System (HDSS), MRC Unit The Gambia at the London School of Hygiene and Tropical Medicine, Banjul, The Gambia; 9 Data Management & Architecture, MRC Unit The Gambia a London School of Hygiene and Tropical Medicine, Banjul, The Gambia; 10 Centre on Climate Change and Planetary Health, MRC Unit The Gambia at The London School of Hygiene and Tropical Medicine, Banjul, The Gambia; 11 Centre for Global Health, Charité Universitatsmedizin Berlin, Berlin, Germany

**Keywords:** Immunisation, COVID-19, Child health, Epidemiology

## Abstract

**Introduction:**

The COVID-19 pandemic caused widespread morbidity and mortality and resulted in the biggest setback in routine vaccinations in three decades. Data on the impact of the pandemic on immunisation in Africa are limited, in part, due to low-quality routine or administrative data. This study examined coverage and timeliness of routine childhood immunisation during the pandemic in The Gambia, a country with an immunisation system considered robust.

**Methods:**

We obtained prospective birth cohort data of 57 286 children in over 300 communities in two health and demographic surveillance system sites, including data from the pre-pandemic period (January 2015–February 2020) and the three waves of the pandemic period (March 2020–December 2021). We determined monthly coverage and timeliness (early and delayed) of the birth dose of hepatitis B vaccine (HepB0) and the first dose of pentavalent vaccine (Penta1) during the different waves of the pandemic relative to the pre-pandemic period. We implemented a binomial interrupted time-series regression model.

**Result:**

We observed no significant change in the coverage of HepB0 and Penta1 vaccinations from the pre-pandemic period up until the periods before the peaks of the first and second waves of the pandemic in 2020. However, there was an increase in HepB0 coverage before as well as after the peak of the third wave in 2021 compared with the pre-pandemic period (pre-third wave peak OR = 1.83, 95% CI 1.06 to 3.14; post-third wave period OR=2.20, 95% CI 1.23 to 3.92). There was some evidence that vaccination timeliness changed during specific periods of the pandemic. Early Penta1 vaccination decreased by 70% (OR=0.30, 95% CI 0.12 to 0.78) in the period before the second wave, and delayed HepB0 vaccination decreased by 47% (OR=0.53, 95% CI 0.29 to 0.97) after the peak of the third wave in 2021.

**Conclusion:**

Despite the challenges of the COVID-19 pandemic, The Gambia’s routine vaccination programme has defied the setbacks witnessed in other settings and remained resilient, with coverage increasing and timeliness improving during the second and third waves. These findings highlight the importance of having adequate surveillance systems to monitor the impact of large shocks to vaccination coverage and timeliness.

**What is already known on this topic**

- Several studies conducted in North America, Europe and Asia showed that the coverage of routine immunisation declined, especially in the early phase of the COVID-19 pandemic.
- While mortality and morbidity from the pandemic were comparatively lower in Africa, data on the impact of the pandemic on routine vaccination are limited, partly due to low-quality routine or administrative data.

**What this study adds**

- We used monthly prospective birth cohort data from over 300 communities in 2 large health and demographic surveillance systems in The Gambia, covering 5 years before and 2 years into the COVID-19 pandemic, to explore 2 important dimensions of immunisation system performance: coverage and timeliness.
- Our findings suggest that the COVID-19 pandemic did not have a significant negative impact on routine vaccination in The Gambia.
- Rather, we observed that coverage and timeliness of vaccinations remained stable in the first year of the pandemic, with significant improvement in both metrics in the second year compared with the pre-pandemic period.

**How this study might affect research, practice or policy**

- Our findings suggest that Gambia’s routine immunisation system was resilient and absorbed the additional shocks imposed by the pandemic.
- Thus, it can be a model for other countries to learn from and adapt strategies to their context in future public health emergencies.

## Introduction

Public health emergencies, such as natural disasters, humanitarian crises, armed conflicts and major disease outbreaks resulting in epidemics and pandemics, can strain country-level health systems and lead to a decline in the provision, demand and utilisation of basic health services.^1–3^ This can worsen the burden of infectious diseases and contribute to increased mortality. For example, during the Ebola outbreak in West Africa from 2014 to 2015, there was a significant reduction in healthcare utilisation, including routine immunisation, especially in regions with a high incidence of Ebola cases.^4 5^ In Liberia and Guinea, the number of children receiving measles vaccinations dropped by 30% and 33%, respectively, following the Ebola outbreak in 2014. This decline was followed by further drops of 25% and 26% in 2015.^5^ The decline in routine immunisation coverage led to an increase in the number of children susceptible to measles and a surge in measles cases that persisted for 2 years following the Ebola outbreak.^5^ The COVID-19 pandemic, which began in December 2019 and caused morbidity and mortality in nearly all countries, also resulted in the biggest setback in routine vaccinations in three decades. In the second year of the pandemic, 18.2 million children globally did not receive the first dose of the diphtheria-tetanus-pertussis containing vaccine, and an additional 6.8 million children were undervaccinated.^6^ These examples show that even a temporary interruption of basic health services, such as routine immunisation, during public health emergencies can lead to secondary health crises. This underscores the importance of monitoring the impact of COVID-19 on routine immunisation. Monitoring can help to identify potentially significant adverse changes and inform the planning of mitigating measures for future similar circumstances.

In addition to the direct effects of the pandemic, such as morbidity and mortality caused by the virus, there are well-documented indirect effects on services like routine immunisation, especially in the initial phase of the COVID-19 pandemic. Such effects have been extensively documented, especially in relation to services such as routine immunisation.^7–12^ They stem from a combination of factors, including the negative impact of the physical measures implemented to reduce COVID-19 infection, such as lockdowns, movement restrictions and the suspension of elective and preventive visits to healthcare facilities. Furthermore, even when medical services are available, people were unable to access them due to transport interruptions, economic hardship and fear of COVID-19 exposure. Healthcare workers may experience similar challenges and concerns, as was evident in the early pandemic phase when access to personal protective equipment was unreliable in many contexts.^13^ These effects are thought to be higher in low-income and middle-income countries with limited healthcare resources and fragile health systems.^7^ Recognising the detrimental effects on routine immunisation services, the WHO promptly issued guidance for sustaining routine immunisation activities as early as March 2020.^14–16^ These guidance strongly recommended that, to the extent feasible and in alignment with local contexts and COVID-19 responses, routine immunisation activities for all eligible individuals should maintain their status as a priority.

Studies examining the impact of the pandemic on routine childhood immunisation gained momentum as early as the first year of the pandemic. A global WHO survey, early in 2020, reported a 70% disruption to routine immunisation services, indicating that services were affected in most countries.^17^ Several studies have been conducted at country level in North America,^8 18 19^ Europe^11 20^ and Asia.^9 21^ A key underlying finding from these studies is a decline in routine immunisation rates, especially in the early phase of the pandemic. This decline is indicated by a drop in vaccine coverage and a considerable decline in routine vaccine ordering by national or regional authorities compared with earlier years. However, in other settings, the evidence has been mixed. For example, in countries such as South Korea, there was little to no effect on routine immunisation.^21^ Another study of South-East Asia and Western Pacific countries found that the impact of the COVID-19 pandemic on routine immunisation was most pronounced in rural and economically disadvantaged communities.^9^ Data for Africa are limited, in part, due to low-quality routine or administrative data. A 2020 study that reported data from 15 African countries found that those with historically high immunisation rates had minimal declines in coverage compared with 2019 rates, while those with lower coverage had larger declines.^22^ This study did not include data from The Gambia, a country with historically high immunisation rates. This is a key evidence gap, as the situation in The Gambia may be different from other high performing, but geographically larger countries such as Senegal and Rwanda, which had been included. Additionally, most of the published studies focused on measuring routine coverage, without examining other important and time-sensitive dimensions of immunisation performance, such as the timeliness of vaccination. Timeliness of vaccination, that is, receiving vaccines within the recommended windows and in an age-appropriate manner,^23^ is essential to achieving the full benefits of vaccines, along with achieving high coverage. Lastly, most of the studies have been based on cross-sectional surveys, which were conducted early in the pandemic, making it difficult to understand and compare the impact on routine vaccination during the later phases of the pandemic.

To address the identified gaps, the aim of this study was to assess the impact of the COVID-19 pandemic on the coverage and timeliness of routine childhood immunisation in The Gambia. To do this, we used routinely collected data from two health and demographic surveillance system (HDSS) sites. We also examined whether the pandemic impacted the coverage and timeliness of vaccination differently across these two regions in The Gambia: one with relatively lower coverage and higher untimely vaccination, and the other with relatively better performance.^24^ We used HDSS data because they offer a unique opportunity to prospectively or prospectively monitor vital statistics and health indicators, including childhood immunisation over a long period of time.^25^ HDSS data are high-quality and population-based, making it an ideal source for studying the impact of the COVID-19 pandemic on routine childhood immunisation. We hypothesise that the COVID-19 pandemic would have led to a statistically significant decrease in routine childhood vaccination coverage and an increase in untimely vaccination (ie, early and delayed vaccinations) in The Gambia. These changes are expected to have been particularly pronounced during the peaks of infections or waves when resources were stretched and disruptions to vaccination services expected to been most severe. We used the birth dose of hepatitis B vaccine (HepB0) and the first dose of pentavalent vaccine (Penta1) as case studies for two reasons. HepB0 is recommended by the WHO to be administered within 24 hours of birth.^26^ Therefore, the uptake and timeliness of HepB0 could be significantly affected by disruptions to delivery of immunisation services caused by the COVID-19 pandemic. On the other hand, the administration of Penta1 is the first-time families interact with the immunisation system after the birth period. Studies indicate that delaying or not receiving Penta1 could have a negative impact on subsequent scheduled doses, creating a cascading effect.^27^

## Methods

### Study context and COVID-19 timeline

**Figure 1:**
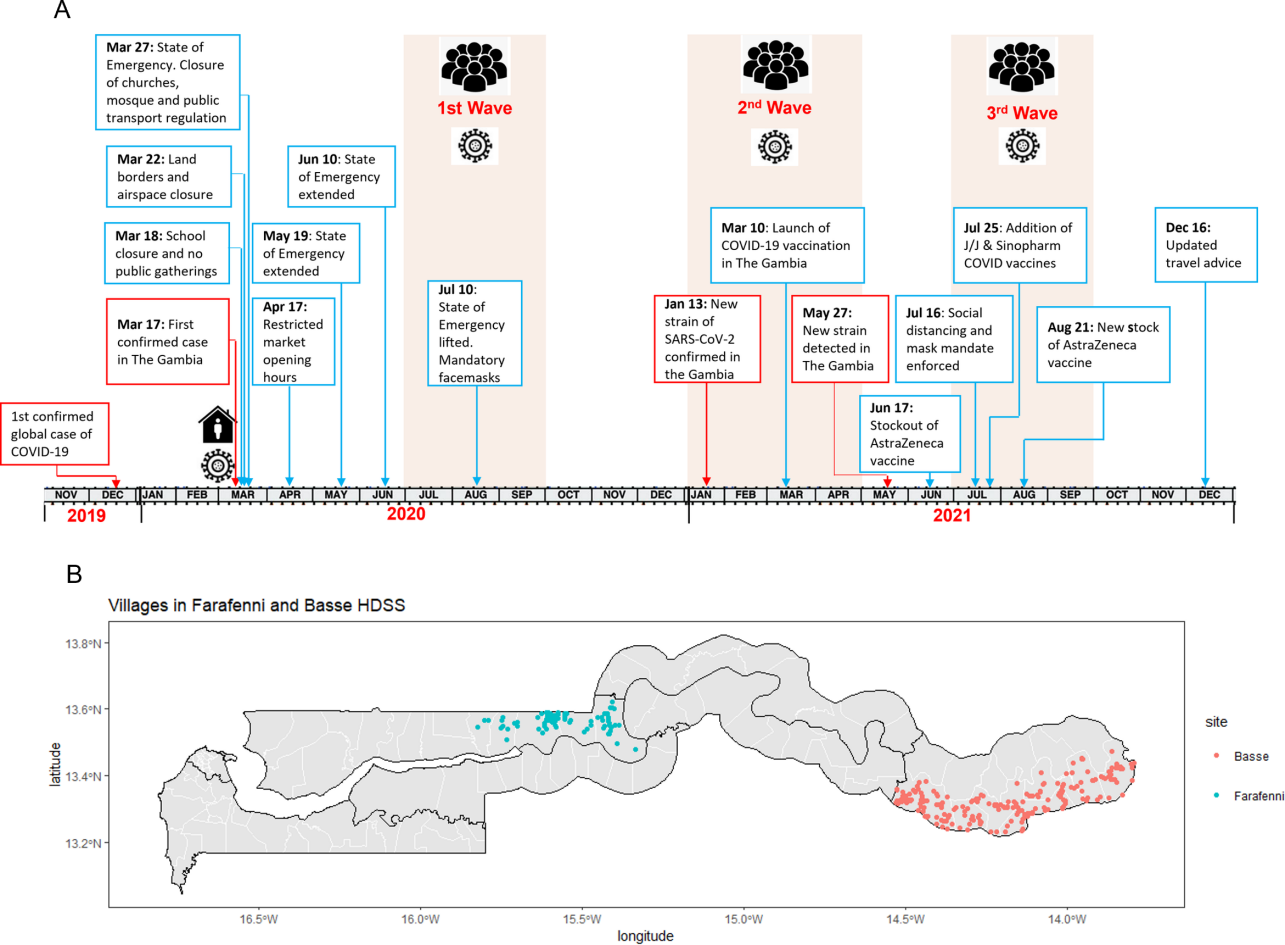
(A) Detailed timeline showing the evolution and measures implemented to control the spread of COVID-19 in The Gambia, March 2020–December 2021. (B) Map of The Gambia showing the location of all the communities covered by the Basse and Farafenni Health and Demographic Surveillance Sites. *In figure 1A, red lines indicate case confirmation, new variants and waves. Blue lines indicate preventive measures implemented by government reduce impact of the pandemic. HDSS, health and demographic surveillance system.

The Gambia, a country located in West Africa, has a population of about 2.5 million people. The median age is 17.8 years, and a national yearly birth cohort of about 90 000 children.^28^ More than half of the population lives in urban areas, mainly on the coast.^29^ The childhood immunisation programme in The Gambia has been remarkably successful, with routine immunisation coverage rates comparable to those of high-income countries. The country has consistently achieved routine coverage of at least 90% for most childhood vaccines for over a decade prior to the COVID-19 pandemic.^30 31^ However, we have previously shown that many children are vaccinated outside of the recommended time frames,^32^ especially in districts in the eastern part of the country.^24^ The first confirmed case of COVID-19 in The Gambia was identified on 17 March 2020.^33^ The evolution of the pandemic and measures taken to control the spread of COVID-19 in The Gambia have been previously described.^34^ In brief, in the days following the confirmation of the first case, the government swiftly implemented a series of measures to curb further transmission. These measures included the prohibition of public gatherings, closure of educational institutions including universities, suspension of air travel, closure of land and sea borders, and closure of non-essential businesses. There were three waves of COVID-19 recorded between March 2020 and December 2021. Figure 1A shows the detailed timeline of the COVID-19 related events in The Gambia.

### Study design

We used an interrupted time-series (ITS) design to examine the impact of the COVID-19 pandemic on the coverage and timeliness of vaccination in a longitudinal cohort of children born 5 years prior to the pandemic and those born within the initial 2 years of the pandemic. Our choice of an ITS design was justified by the availability of sequential, equally spaced measurements of vaccination coverage and timeliness before and after the COVID-19 pandemic’s interruption. This design, with its substantial number of time points, provided a robust framework for isolating the pandemic’s specific effect.^35^

### Data sources

We used data from the Basse and Farafenni Health and Demographic Surveillance Systems (BHDSS and FHDSS henceforth), which were established about four decades ago. BHDSS and FHDSS are located in eastern and central parts of The Gambia and prospectively follow-up a combined population of 280 000 persons in about 9000 households in over 300 communities (figure 1B).^36^ The BHDSS is predominantly rural and located in the part of The Gambia with comparatively lower vaccination coverage and higher rates of delayed vaccination.^24^ The FHDSS is predominantly peri-urban and has relatively better coverage and timeliness. The yearly birth cohort is approximately 9000 children in both HDSS. Both sites have supported cutting-edge medical, public health and demographic research since their inception.

Detailed information about the design and methodology of the BHDSS and FHDSS have been described elsewhere,^37^ and in online supplemental material. In brief, BHDSS and FHDSS conduct routine surveillance rounds every 4 months to collect health and demographic data from all consenting households in all HDSS communities. Every child born within the HDSS communities is automatically enrolled and followed up by fieldworkers. Information on the date of birth and date of vaccinations is extracted from parent-held vaccination cards during each census round. Any missing information is routinely updated in subsequent rounds for all individuals who have been enrolled. This approach makes HDSS data more robust and potentially better for our purpose than cross-sectional population surveys which although often have high geographical coverage but do not allow for longitudinal follow-up and additionally rely on potentially biased information from caregiver recall to evaluate vaccination coverage.

10.1136/bmjgh-2023-014225.supp1 Supplementary data

### Data processing

To synthesise adequate evidence regarding the monthly trend in coverage and timeliness well before the pandemic, we included data for 7 years, that is, from all children born from 1 January 2015 to 31 December 2021 in all FHDSS and BHDSS households. The decision to include data for 5 years before and 2 years during the pandemic was also to balance out temporal confounding factors, such as seasonal variations (wet and dry seasons) and monthly birth rate variations. We defined the pre-pandemic epoch as the period from January 2015 to February 2020. The pandemic epoch started in March 2020, when the first case of COVID-19 was confirmed in The Gambia. The time series ends in December 2021. Subsequently, we created 84 birth cohorts, each corresponding to children born in a specific month, starting from January 2015 (cohort 1) to December 2021 (cohort 84). The outcome variable was vaccination coverage and timeliness of vaccination among each of these monthly birth cohorts. Detailed information about the number of eligible children per month and those excluded due to improbable vaccination dates is shown in the online supplemental material

### Defining and computing vaccination coverage and timeliness

We defined vaccination coverage as the monthly proportions of children who received the vaccine of interest (HepB0 or Penta1) relative to the respective monthly birth cohorts, regardless of timing. Timeliness was determined based on the accepted vaccination window in The Gambia,^38^ in line with recent timeliness studies from The Gambia.^24 32^ Age at vaccination (in days) for each vaccine was calculated by finding the difference between vaccination and birth dates for every child. Timely HepB0 and Penta1 was defined as vaccination within 24 hours of birth and between 2 and 3 months of age (ie, 61–90 days), respectively, in accordance with the national vaccination schedule in The Gambia.^38^ For children born in BHDSS from September 2019 until December 2021, timely Penta1 was considered as vaccination between 6 and 10 weeks of age (42–70 days). This modified definition for Penta1 in BHDSS was adopted due to the ongoing prospective, cluster-randomised, non-inferiority field trial of an alternative schedule for one dose of pneumococcal conjugate vaccines in this area from September 2019.^39^ This trial, conducted in collaboration between the MRC Unit The Gambia at LSHTM and the Gambian Ministry of Health, administers Penta1 at 6 weeks instead of the usual 2 months. HepB0 and Penta1 vaccinations that were received after the accepted window were considered delayed, whereas Penta1 vaccinations that were received before the accepted window were considered early.

### Modelling counterfactual scenario and testing changes due to the pandemic

We performed one-step-ahead simulations to generate the counterfactual scenario after the onset of the pandemic (from cohort 63 or March 2020) using a binomial first-order autoregressive (AR1) time series regression model as shown below in equation (1). This model can be fitted without the $\beta_{1}t$ term, but this trend term was included to explicitly test for an overall increasing or decreasing trend in the data. Also, for delayed HepB0, the model that included the explicit trend term performed slightly better than the model without it. The model is given by

(1) $$Y_{t}∼Binomial\left(N_{t},p_{t}\right),t=1,\ldots ,n=84,\;logit\left(p_{t}\right)=\beta_{0}+\beta_{1}t+\omega_{t},\;\omega_{t}|\omega_{t-1}∼N\left(\rho \omega_{t-1},\sigma^{2}\right),\;\omega_{1}∼N\left(0,\frac{\sigma^{2}}{1-\rho^{2}}\right),$$

where $Y_{t}$ is the number of vaccinated children out of a birth cohort of size $N_{t}$ at time t , $p_{t}$ is the corresponding underlying true vaccination coverage, $\beta_{0}$ (intercept) and $\beta_{1}$ are regression coefficients and $\omega_{t}$ is an AR(1) term with autoregressive parameter, ρ, and conditional variance, $\sigma^{2},$ accounting for residual serial correlation.

To estimate changes (ie, level change and change in slope) in vaccination coverage and timeliness during the peaks of infections (waves), we extended the base model in (1) to an ITS model.^40^ The variable of interest was the proportion of timely, delayed, or early vaccination per month. We assessed changes in the time periods *before* the peaks of the first (𝑇_1_ = 63, April–August 2020), second (𝑇_2_ = 68, September 2020–March 2021), and third (𝑇_3_ = 75, April–August 2021) waves of the pandemic, *as well as* the period *after* the peak of the third wave (𝑇_4_ = 80, September–December 2021), relative to the pre-pandemic period (ie, from January 2015 to March 2020). We coded the level changes as indicator variables (ie, $D_{t63},D_{t68},D_{t75},D_{t80}$ ), with each variable representing a given time period during which changes are evaluated. For example, $D_{t63}$ is used to assess a level change between the start of the pandemic in The Gambia in April 2020 and the peak of the first wave in August 2020 and is coded 1 within this time period and 0 elsewhere.

Slope changes, assessed using the terms [*t−T*_1_]*D*_*t*63_, [*t−T*_2_]*D*_*t*68_, [*t−T*_3_]*D*_*t*75_ and [*t−T*_4_]*D*_*t*80_, were coded as sequentially numbered months during each time period, and 0 before or after.

The baseline monthly trend in coverage and timeliness (time) was coded sequentially throughout the entire study period. The ITS model with a binomial likelihood can be written as

(2) $$\begin{array}{rl} & Y_{t}∼\mathrm{Binomial}\left(N_{t},p_{t}\right),t=1,\ldots ,n=84\\ & \mathrm{logit}\left(p_{t}\right)=\beta_{0}+\beta_{1}t+\beta_{2}D_{t63}+\beta_{3}\left[t-T_{1}\right]D_{t63}+\beta_{4}D_{t68}\\ & +\beta_{5}\left[t-T_{2}\right]D_{t68}+\beta_{6}D_{t75}\\ & +\beta_{7}\left[t-T_{3}\right]D_{t75}+\beta_{8}D_{t80}+\beta_{9}\left[t-T_{4}\right]D_{t80}+\omega_{t},\\ & \omega_{t}|\omega_{t-1}∼N\left(\rho \omega_{t-1},\sigma^{2}\right),\\ & \omega_{1}∼N\left(0,\frac{\sigma^{2}}{1-\rho^{2}}\right), \end{array}$$

where the regression coefficient $\beta_{0}$ estimates the pre-pandemic intercept and $\beta_{1}$ —the pre-pandemic slope. The regression coefficients $\beta_{2},\beta_{4},\beta_{6},\beta_{8}$ are intercept terms measuring immediate level changes in the coverage and timeliness indicators within the segments following the pandemic, and $\beta_{3},\beta_{5},\beta_{7},\beta_{9}$ measure corresponding changes in slope. As in model (1), $\omega_{t}$ is an AR1 random effect used to capture residual autocorrelation in the model. Both models (1) and (2) were implemented using the integrated nested Laplace approximation approach, in a fully Bayesian framework.^41^ We report the ORs and corresponding credible intervals (CIs) of all the regression coefficients.

To provide a comprehensive understanding of delayed vaccination, we calculated the mean number of days children in each birth cohort were delayed for HepB0, in addition to the monthly proportion of delayed vaccination. This mean delay was then compared against the overall average delay for HepB0 vaccination across the entire population and between the pre-pandemic and pandemic periods. All analysis was done in R (R Development Core Team, 2023). We report and compare findings for vaccination coverage, proportion delayed, mean number of days delayed and proportion with early vaccination for the monthly birth cohorts in the pre-pandemic and pandemic periods.

## Results

From January 2015 to December 2021, a total of 57 286 children were born in the Basse and Farafenni HDSSs and were eligible for HepB0 and Penta1 vaccination. This number includes 43 428 children from villages within the Basse HDSS and an additional 13 858 children from the Farafenni HDSS. Overall, the coverage of HepB0 vaccination was generally higher than that of Penta1 vaccination throughout the study duration. The proportion of children with delayed HepB0 vaccination was also higher than that of delayed Penta1 vaccination.

### Coverage of HepB0 and Penta1

**Figure 2:**
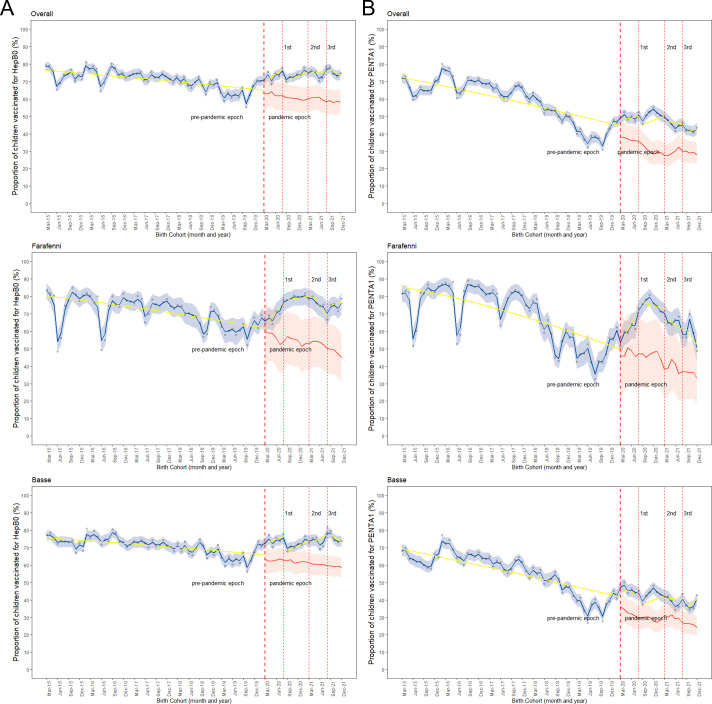
Observed hepatitis B vaccine (HepB0) (A) and pentavalent vaccine (Penta1) (B) coverage, counterfactual scenario and changes (level and slope) due to the pandemic overall, in Farafenni and Basse. *Red-dotted lines indicate when the first case of COVID-19 was confirmed in The Gambia, the peaks of the first, second and third waves. Blue line indicates observed coverage and 95% credible intervals; red line=counterfactual scenario and 95% credible interval; yellow line indicates the change in slope for the proportion of children vaccinated.

Figure 2A and B illustrate the coverage of HepB0 and Penta1 for monthly birth cohorts during the pre-pandemic and pandemic epochs. The observed HepB0 and Penta1 vaccination coverage declined over time in the pre-pandemic period, but an increasing trend was observed during the pandemic period, compared with the counterfactual scenario as shown in figure 2A and B.

Overall, the binomial regression model did not find statistically significant differences in the coverage of HepB0 and Penta1 vaccinations between the pre-pandemic period and the period just before the peaks of the first, second, and before and after the third waves of the pandemic, based on level changes. The only exception was the period before and after the peaks of the third wave for HepB0 (table 1 and figure 2A). The likelihood of receiving HepB0 vaccination increased by 83% (OR=1.83, 95% CI 1.06 to 3.14) and 120% (OR=2.20, 95% CI 1.23 to 3.92) in the period before and after the peaks of the third wave, respectively, compared with the pre-pandemic period (table 1). The changes were similar in Farafenni and Basse (figure 2A). In Farafenni, the likelihood of receiving HepB0 increased by 150% (OR=2.54, 95% CI 1.14 to 11.2) during the period preceding the third wave’s peak. In the Basse area, there was an increase of 120% (OR=2.21, 95% CI 1.24 to 3.89) following the third wave’s peak, compared with the pre-pandemic period (online supplemental table). No statistically significant changes in the slope of the trends were observed overall, or in Farafenni and Basse.

**Table 1:** Parameter estimates for the likelihood of change in coverage and the proportion of delayed and early hepatitis B vaccine (HepB0) and pentavalent vaccine (Penta1) vaccinations in the pre-pandemic and pandemic periods in The Gambia*

	HepB0			Penta1		
Coverage	Estimate/OR	95% credible interval		Estimate/OR	95% credible interval	
Level change						
Before first wave	1.24	0.79	1.93	1.15	0.75	1.74
Before second wave	1.28	0.79	2.01	1.10	0.53	2.19
Before third wave	1.83	1.06	3.14	1.41	0.54	3.41
After third wave	2.20	1.23	3.92	1.17	0.40	3.11
Change in slope						
Pre-pandemic	0.99	0.99	1.00	0.98	0.97	1.00
Before first wave	1.08	0.95	1.23	1.04	0.89	1.20
Before second wave	1.06	0.96	1.15	1.05	0.94	1.18
Before third wave	1.01	0.88	1.16	1.00	0.86	1.17
AFTER third wave	0.95	0.80	1.14	1.05	0.87	1.25
${\hat{\sigma }}^{-2}$	31.56	17.99	49.37	11.12	3.74	21.63
$\hat{\rho }$	0.50	0.25	0.72	0.87	0.75	0.96
Delayed						
Level change						
Before first wave	1.25	0.73	2.12	1.60	0.79	3.41
Before second wave	1.65	0.99	2.67	1.13	0.30	3.29
Before third wave	1.46	0.82	2.59	0.96	0.18	3.25
After third wave	**0.53**	**0.29**	**0.97**	0.97	0.16	3.61
Change in slope						
Pre-pandemic	**0.98**	**0.97**	**0.99**	1.00	0.99	1.03
Before first wave	1.05	0.90	1.23	0.90	0.70	1.13
Before second wave	0.94	0.86	1.04	0.94	0.79	1.11
Before third wave	0.81	**0.70**	0.94	0.96	0.76	1.21
AFTER third wave	1.09	0.90	1.32	0.92	0.69	1.22
${\hat{\sigma }}^{-2}$	32.25	15.71	60.57	7.21	2.52	13.96
$\hat{\rho }$	0.29	−0.20	0.70	0.78	0.53	0.94
Early						
Level change						
Before first wave				0.54	0.19	1.60
Before second wave				0.30	0.12	0.78
Before third wave				0.49	0.16	1.56
After third wave				0.48	0.14	1.72
Change in slope						
Pre-pandemic				0.99	0.99	1.00
Before first wave				1.01	0.76	1.34
Before second wave				1.12	0.93	1.34
Before third wave				0.99	0.72	1.36
After third wave				0.92	0.60	1.42
${\hat{\sigma }}^{-2}$				9.95	5.63	15.87
$\hat{\rho }$				0.38	0.07	0.66

This table summarises the overall estimates. See the online supplemental material for Farafenni-specific and Basse-specific parameter estimates.

### The proportion of delayed HepB0 and Penta1

**Figure 3:**
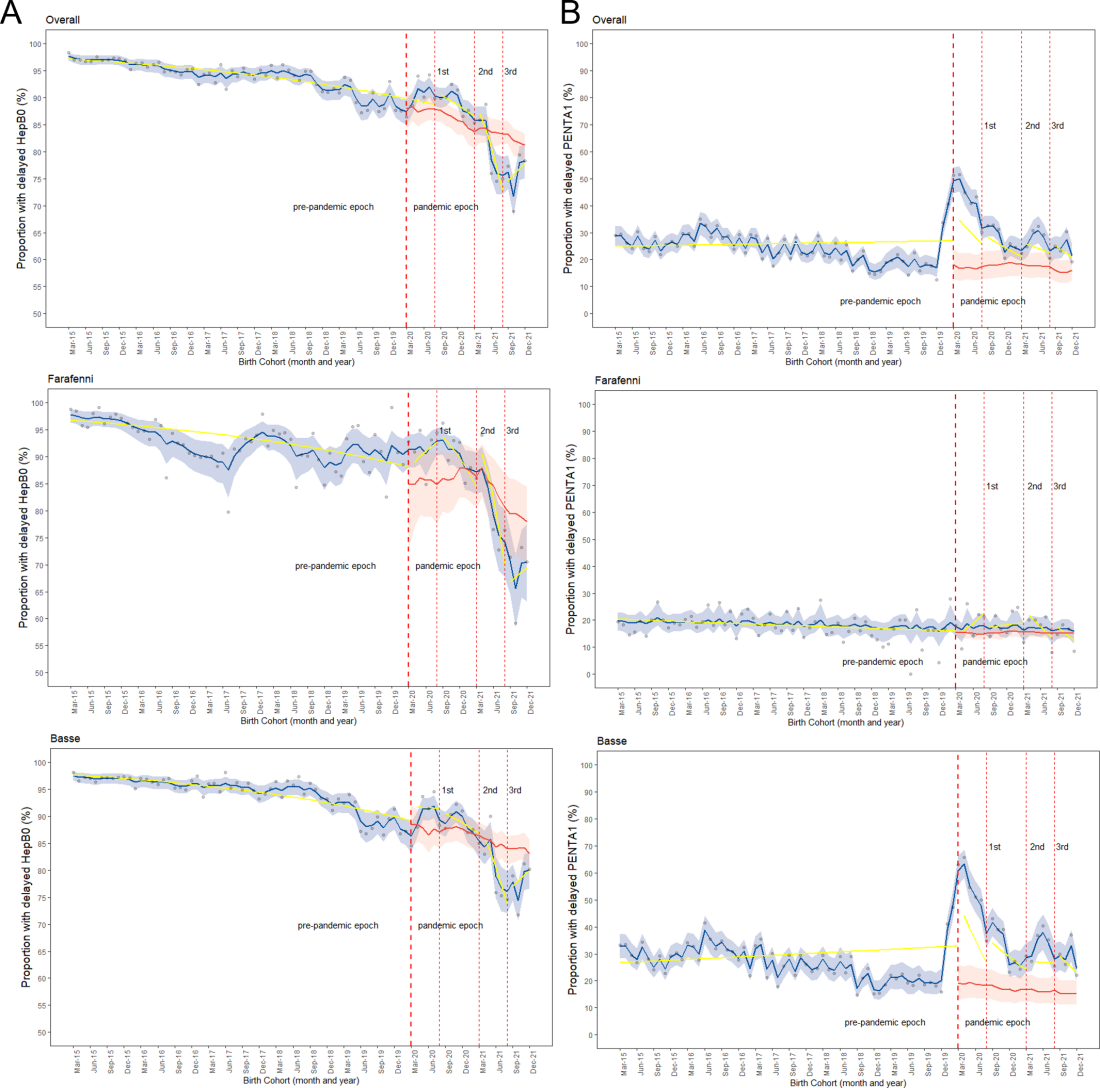
Observed hepatitis B vaccine (HepB0) (A) and pentavalent vaccine (Penta1) (B) delayed vaccination, counterfactual scenario and changes (level and slope) due to the pandemic overall, in Farafenni and Basse. *Red-dotted lines indicate when the first case of COVID-19 was confirmed in The Gambia, the peaks of the first, second and third waves; blue line indicates observed coverage and 95% credible intervals; red line=counterfactual scenario and 95% credible interval; yellow line indicates the change in slope for the proportion of children vaccinated.

Overall, there was a downward trend in observed delayed HepB0 vaccination in the pre-pandemic period. This trend in delayed HepB0 plateaued in the first year of the pandemic, before a rapid decline in the second year of the pandemic period, compared with the counterfactual scenario (figure 3A). Delayed Penta1 was generally stable (ranging from 20 to 35%) in the pre-pandemic period, with a rapid rise in the months leading up to the pandemic, entirely driven by data from the BHDSS area. However, the monthly proportion of observed delayed Penta1 steadily declined over time (figure 3B).

There were no statistically significant differences in the proportions of delayed HepB0 and Penta1 vaccinations between the pre-pandemic period and the period before the peaks of the first, second, and before and after the third waves of the pandemic, based on level changes (table 1 and figure 3A and B). The only exception was the period after the peaks of the third wave for HepB0 (figure 3A), where the likelihood of delayed vaccination decreased by 47% (OR=0.53, 95% CI 0.29 to 0.97). This finding is consistent with level changes in Farafenni and Basse, where no statistically significant differences were found in the proportion of delayed HepB0 and Penta1 vaccinations between the pre-pandemic period and the waves of infections in the pandemic period, based on level change (online supplemental table). Regarding the change in slope of the trend for delayed HepB0, there was a statistically significant decrease in the pre-pandemic period overall and in Basse. Similarly, there was a statistically significant decrease in the slope of the trend for delayed HepB0 for the period before the peak of the third wave, compared with the pre-pandemic period. Overall, the odds of delayed HepB0 decreased by 19% (OR=0.81, 95% CI 0.70 to 0.94) and in Farafenni by 28% (OR=0.72, 95% CI 0.54 to 0.95). The change in slope for Penta1 was not statistically significant (table 1).

### Number of days delayed for HepB0

**Figure 4:**
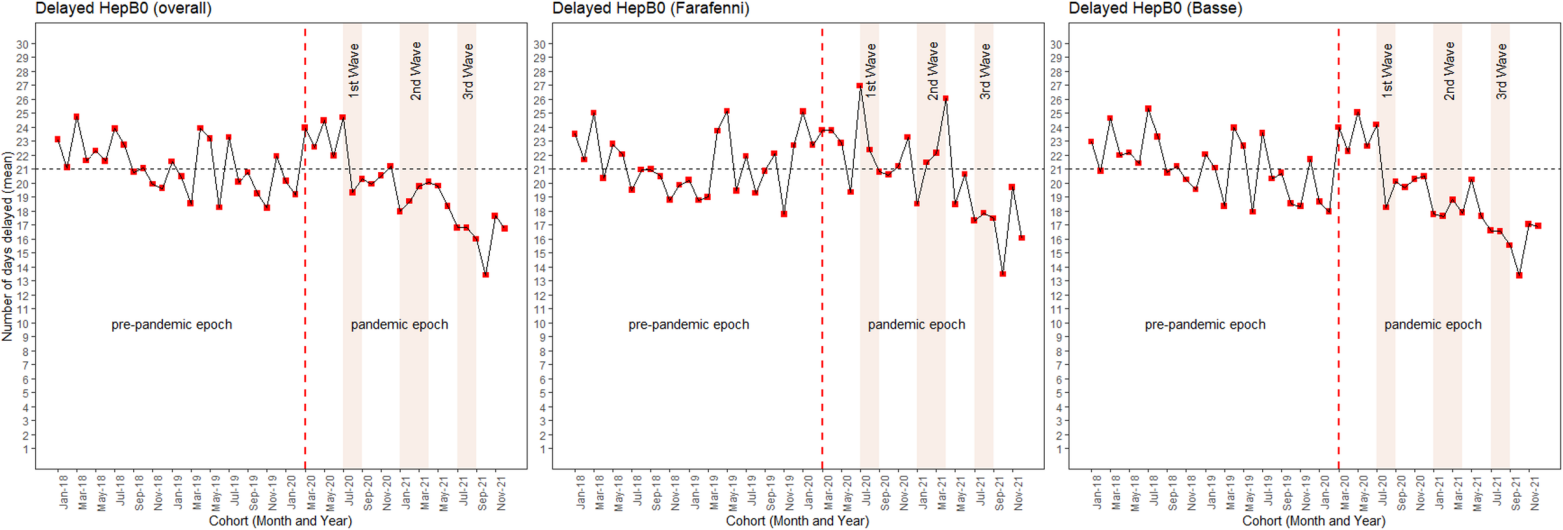
Observed mean number of days with delayed hepatitis B vaccine (HepB0) vaccination per monthly birth cohort in the pre-pandemic compared with the pandemic period overall, in Farafenni and Basse.

The overall mean number of days with delayed HepB0 was 21 days. In the pre-pandemic period, the monthly mean number of days with delayed HepB0 fluctuated above and below this overall mean. Before the first wave of the pandemic (March–July 2020), the monthly mean number of days delayed for HepB0 was generally above 21 days. However, this gradually decreased below 21 days and has remained so since September 2020 (figure 4). This pattern was mirrored in Basse. In Farafenni, the pattern in the pre-pandemic and pandemic periods was not different.

### The proportion of early Penta1

**Figure 5:**
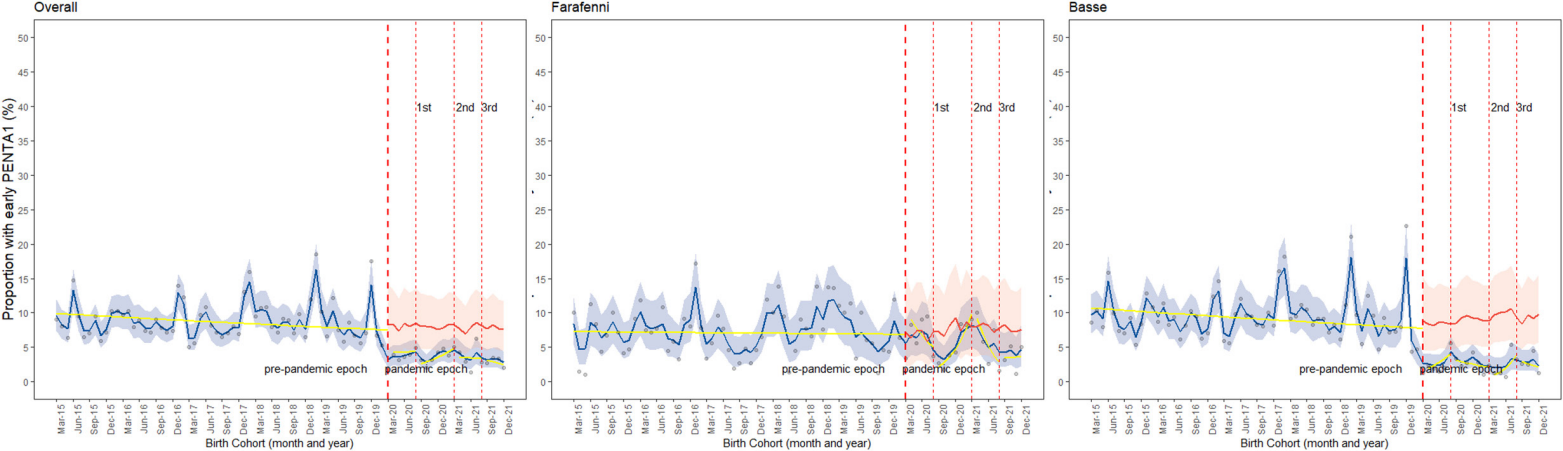
Observed hepatitis B vaccine (HepB0) (A) and pentavalent vaccine (Penta1) (B) early vaccination, counterfactual scenario, and changes (level and slope) due to the pandemic overall, in Farafenni and Basse. *Red-dotted lines indicate when the first case of COVID-19 was confirmed in The Gambia, the peaks of the first, second and third waves; blue line indicates observed coverage and 95% credible intervals; red line=counterfactual scenario and 95% credible interval; yellow line indicates the change in slope for the proportion of children vaccinated.

Overall, the trend in the observed monthly proportion of early Penta1 vaccination was stable throughout the pre-pandemic period. This trend was also stable in the pandemic period, but lower when compared with the counterfactual scenario (figure 5). There were statistically significant differences in the observed proportion of early Penta1 vaccinations between the pre-pandemic period and the period before the peaks of the second wave of the pandemic period, both overall and in Farafenni, based on level changes (table 1 and online supplemental table). Compared with the pre-pandemic period, the likelihood of early Penta1 vaccination decreased by 70% (OR=0.30, 95% CI 0.12 to 0.78) and 77% (OR=0.23, 95% CI 0.06 to 0.85) in the period before the peaks of the second wave, overall and in Farafenni, respectively (table 1). Similarly, significant decreases in the proportion of early Penta1 vaccinations were observed between the pre-pandemic period and the periods before the peaks of the first (OR=0.20, 95% CI 0.05 to 0.76) and third (OR=0.09, 95% CI 0.02 to 0.47) waves of the pandemic period in Basse (online supplemental table). No statistically significant changes in the slope of the trends were observed overall, nor in Farafenni and Basse (online supplemental table).

## Discussion

Our study aimed to determine if there were any changes in vaccination coverage and timeliness in The Gambia during the COVID-19 pandemic, compared with before the pandemic. We hypothesised that the COVID-19 pandemic led to a decrease in coverage and an increase in *untimely vaccination* (ie, delayed and early) for subsequent monthly birth cohorts during the pandemic in The Gambia. We found no support for this hypothesis. Rather, our analysis showed that overall, there was no significant change in the coverage of HepB0 and Penta1 vaccinations in the period before the peaks of the first and second waves of the pandemic compared with the pre-pandemic period. These findings diverge from our initial hypothesis, which had anticipated a significant decrease in coverage and an increase in delayed and early vaccination during the pandemic. The findings also differ from previous studies which reported a significant decline in routine vaccination coverage and delays due to the pandemic.^8 10 11 18 20 42^ Nonetheless, our findings are consistent with reports showing that African countries with similarly high pre-pandemic immunisation coverage, such as Senegal, Rwanda and Eritrea, have managed to maintain these levels.^22^ This is also similar to data from South Korea, which showed that there was little to no effect on routine immunisation due to the pandemic.^21^

Our findings suggest that the Gambia’s routine immunisation system was resilient and absorbed the additional shocks imposed on it by the pandemic. This is evident in the maintenance of coverage and timeliness in the first year, and the actual increase in coverage and decrease in delayed and early vaccination in the second year. There are several plausible explanations for these observed findings. The Gambia developed and implemented mitigation strategies to reduce the impact of the pandemic on essential health services. In March 2020, just after the country confirmed its first case of COVID-19, the Ministry of Health developed a guideline for maintaining essential services, including immunisation.^43^ This guideline prioritised routine childhood vaccination, specifically, birth dose vaccination, the next dose at 2 months and other subsequent doses.^43^ It also mandated the screening and referral for vaccination of eligible children during visits for other services, the continuation of VPD surveillance, and enhanced community sensitisation about the need to continue all scheduled routine vaccinations. The guideline mandated the continuation of immunisation delivery at outreach vaccination sites,^43^ a key strategy for delivering routine vaccines that have contributed to the success of the immunisation programme in The Gambia.^44^ To reduce waiting time and avoid overcrowding at clinics, some ancillary activities were temporarily suspended during vaccination activities conducted in health facilities (fixed-clinics) until late 2020.^45^ These activities included child weight measurement and updating of daily records logbooks, except for recording information on hand-held vaccination cards. Furthermore, in July 2020, before the first wave of the pandemic in The Gambia, the immunisation programme also carried out intensive community sensitisation. They held radio programmes and visited communities to dispel rumours and provide answers to community members’ questions about COVID-19. Lastly, the Gambian Expanded Programme on Immunisation (EPI) borrowed routine vaccines from neighbouring Senegal in anticipation of logistical challenges that might deplete their stock. Taken together, these activities likely ensured the maintenance of adequate supply of services and uptake of routine vaccinations during the pandemic.

Our findings are further strengthened by the fact that the mean number of days monthly birth cohorts of children were delayed for HepB0 continuously declined after the onset of the first wave of the pandemic (July 2020 onward) and remained well below the overall mean of 21 days throughout the pandemic period. The WHO target is to ensure all children receive timely HepB0 within 24 hours of birth.^26^ However, a consistent decline below 21 days despite the pandemic is a notable improvement, as the mean days delayed for HepB0 historically fluctuated around the overall mean of 21 days before the pandemic. Several factors may explain why the delivery of HepB0 improved despite the pandemic. First, about 2 years prior to the pandemic, The Gambia launched a major initiative to improve the timeliness of HepB0 administration, as hepatitis B virus infection remains endemic in the country, with 15%–20% of the population chronically infected despite high coverage.^46^ This initiative strengthened the administration of HepB0 in all health facilities where deliveries occurred by assigning specific health workers to administer HepB0 within the first day of birth. Second, the low number of confirmed COVID-19 cases in the study area compared with urban coastal regions,^47^ as well as the lax enforcement of the government stay-at-home order in the study area,^45^ may have resulted in a low degree of risk perception. As a result, families may have maintained their health-seeking behaviours related to facility delivery and subsequent receipt of HepB0. Third, the government mandate to prioritise birth doses of vaccines and continue vaccination delivery at outreach vaccination sites during the pandemic could have ensured that services were delivered in a timely manner and available throughout this period.^43^ Fourth, in January 2021, the Gambian EPI implemented an electronic immunisation register system called ‘*MyChild* Solution’ across the country.^48^ One of the key features of the *MyChild* Solution is the ability to autogenerate predefined indicators and send out SMS messages to valid phone numbers of parents registered in the system. The EPI programme leveraged the monitoring potential of the solution and, since January 2020 when the system was still being pilot tested, introduced a HepB0 vaccination timeliness indicator which is monitored monthly at the health facility level.^48^ Through this solution, facilities throughout the country could monitor timeliness of HepB0 and take data-driven decisions.

Our findings differ in some respects from those of the only other study from The Gambia that has so far explored the impact of the pandemic on vaccination services delivery. In contrast to our findings, the previous study reported reduced clinic visits and vaccination doses administered, particularly for birth doses, for only 3 months after the onset of the pandemic in The Gambia compared with the baseline period.^45^ Although the previous study also reported data from BHDSS, which is one of our study sites, slight differences in the objectives and methodology of the two studies could explain the differences in findings. For example, the previous study’s outcome measure was the monthly number of clinic attendances and vaccines administered, which may be correlated but different from our study’s outcome measure of coverage and timeliness (early and delayed) for each monthly birth cohort. Although the previous study may have found that reported monthly clinic attendance and vaccine doses administered declined, our definition of coverage was based on the cohort of children born for each month. This means that even if monthly visits were briefly reduced in the initial phase of the pandemic, children could still have received doses after their scheduled doses, even outside of their birth month. Furthermore, the previous study was relatively short in duration, as it included data covering only 7 months before the pandemic and 9 months afterwards. Our study included data covering 5 years before the pandemic and 2 years into the pandemic (84 months), and we compared the outcome variables with the temporal trends of multiple years. Due to its short duration, the previous study may not have adequately accounted for confounding factors due to temporal trends occasioned by seasons (wet and dry seasons) and monthly variations in birth rates, unlike our study. Lastly, unlike our study, the previous study did not assess changes in timeliness. Therefore, we cannot ascertain from their data if the decline in clinic visits translated into delayed vaccination or not. Aside from the difference in findings already discussed, the previous study reported that clinic visits and doses administered returned to pre-pandemic levels after a brief decline, which is consistent with our findings. Additionally, the brief decline in clinic visits could explain the brief but rapid rise in the monthly delayed Penta1 vaccination, which was driven entirely by data from BHDSS, their study location.

In our study, we also aimed to understand whether the pandemic impacted the coverage and timeliness of vaccination differently in the BHDSS area (with relatively lower coverage and higher untimely vaccination) compared with the FHDSS area (with relatively better performance). Aside from the brief but rapid rise in monthly delayed Penta1 vaccination in the BHDSS area and the peaks of mean delay for HepB0 above 21 days for monthly birth cohorts observed almost throughout 2020 in the FHDSS area, the impact of the pandemic on coverage and early vaccination was similar in both areas. The minimal difference in the impact of the pandemic in both areas, despite baseline data showing differences in coverage and timeliness in both locations,^24^ likely indicates that mitigation measures were implemented in a way that ensured immunisation services in both locations were not negatively impacted by the pandemic.

Our study has some limitations. HDSS communities are observed longitudinally, and households participate in multiple studies where they seldom receive interventions, including vaccinations. This might make them not representative of the general population. Additionally, some individuals or households within the HDSS communities might modify their behaviour (eg, vaccination uptake) because they are aware that they are within a surveillance system—the *Hawthorne effect*.^49^ Despite these limitations, our study has several strengths. First, unlike most previous studies, which were based on cross-sectional surveys or electronic immunisation registers, we used routine surveillance data from two large HDSSs. HDSS datasets offer several advantages, including temporal coverage, coverage of underdocumented or often missed communities, and the ability to conduct detailed linkage.^50^ Electronic immunisation registers typically only cover individuals who visit immunisation clinics, so they miss out on subpopulations that are unvaccinated or have not interfaced with facilities. HDSSs, on the other hand, conduct a total population census of entire communities, so our findings are likely to reflect the true situation of coverage. Another strength of our study is that our dataset covers multiple years before the pandemic and 2 years into the pandemic. This increases the validity of our findings, as our dataset accounted for temporal trends in seasons, birth rates and other factors that might confound coverage and timeliness estimates. Finally, our study examined both vaccination timeliness and coverage for vaccines given early in infancy. This approach is significant because vaccination timeliness is sensitive to disruptions in services, and studies have shown that not receiving or delaying earlier childhood vaccine doses can potentially initiate a cascading effect, impacting subsequent scheduled doses.^27^ While we did not examine doses given in later infancy, we can likely extrapolate the likely impact due to the fact that we used sensitive markers (timeliness and birth doses of vaccines). We do not anticipate widely varying outcomes, given that the only previous study from The Gambia showed that clinic visits for vaccines given in later infancy were minimally impacted.^45^

## Data Availability

Data are available upon reasonable request. Data will be made available on reasonable request through the corresponding author.
