# Supplementary material for: Impact of the COVID-19 pandemic on the coverage and timeliness of routine childhood vaccinations in the Gambia, 2015–2021
Source: BMJ Glob Health. 2023 Dec 26;8(12):e014225. doi: 10.1136/bmjgh-2023-014225 (PMC10753753; doi:10.1136/bmjgh-2023-014225)
Supplement: Supplementary data [file bmjgh-2023-014225supp001.pdf]

Supplementary material

Impact of the COVID-19 pandemic on the coverage and timeliness of routine childhood vaccinations in The Gambia, 2015 – 2021

Oghenebrume Wariri, Chigozie Edson Utazi, Uduak Okomo, Alieu Sowe, Malick Sogur, Sidat Fofana, Esu Ezeani, Lamin Saidy, Golam Sarwar, Bai-Lamin Dondeh, Kris A Murray, Chris Grundy, Beate Kampmann

Contents

Additional information on HDSS data collection 2

Monthly birth cohort of children included per month, 2015 – 2021 (overall) 3

Monthly birth cohort of children included per month, 2015 – 2021 (Basse) 4

Monthly birth cohort of children included per month, 2015 – 2021 (Farafenni) 5

Reasons and numbers of children excluded from the analysis 6

Parameter estimates for change in vaccination in Basse HDSS 7

Parameter estimates for change in vaccination in Farafenni HDSS 8

Extra Results: Changes in Penta1 timely vaccination due to the pandemic and counterfactual scenario overall (i.e., Basse and Farafenni HDSS combined) 9

Extra Results: Changes in Penta1 timely vaccination due to the pandemic and counterfactual scenario in Basse HDSS area 10

Extra Results: Changes in Penta1 timely vaccination due to the pandemic and counterfactual scenario in Farafenni HDSS area 11

Wariri O, *et al. BMJ Glob Health* 2023; 8:e014225. doi: 10.1136/bmjgh-2023-014225

Additional information on HDSS data collection

Individuals enter the surveillance population of the Health and Demographic Surveillance System (HDSS) through three main pathways: initial enumeration, birth within the surveillance area, or in-migration. Fieldworkers visit every compound under surveillance at least once every quarter to update the survival and residency status of all residents. These visits are supplemented by information from resident village reporters, volunteers trained to record demographic events within their villages. This data is then cross-checked with information recorded in each household's registration book (HRB), which contains details of all household members and past events. Fieldworkers use these HRBs to interview household heads every four months, verifying and updating the status of individual members. This comprehensive data collection process allows for the recording of deaths, births, migration within or beyond the surveillance area, health-related data, including routine childhood immunization records. Electronic Data Capture was implemented since 1st October 2015 in Farafenni HDSS (FHDSS), and 1st March 2016 in Basse HDSS (BHDSS).

Wariri O, *et al. BMJ Glob Health* 2023; 8:e014225. doi: 10.1136/bmjgh-2023-014225

Monthly birth cohort of children included per month, 2015 – 2021 (overall)

Table S1: Detailed information about the number of eligible children per month from the two Health and Demographic Health System in The Gambia (overall), 2015 – 2021

	Years included in the analysis and eligible children						
**Months**	**2015**	**2016**	**2017**	**2018**	**2019**	**2020**	**2021**
January	829	753	803	714	805	873	723
February	670	641	667	637	755	776	573
March	659	594	679	682	790	678	556
April	669	488	607	637	572	637	482
May	635	498	531	598	650	649	528
June	773	596	579	511	541	552	503
July	649	457	502	435	485	544	425
August	596	513	552	615	634	733	461
September	775	769	747	884	869	911	695
October	782	800	877	1021	932	965	758
November	744	803	795	966	856	851	660
December	676	744	685	839	757	766	665
**Total**	**8457**	**7656**	**8024**	**8539**	**8646**	**8935**	**7029**

Overall, 57,286 children were included in this analysis spanning 2015 – 2021 (i.e., 5 years before and 2 years into the pandemic)

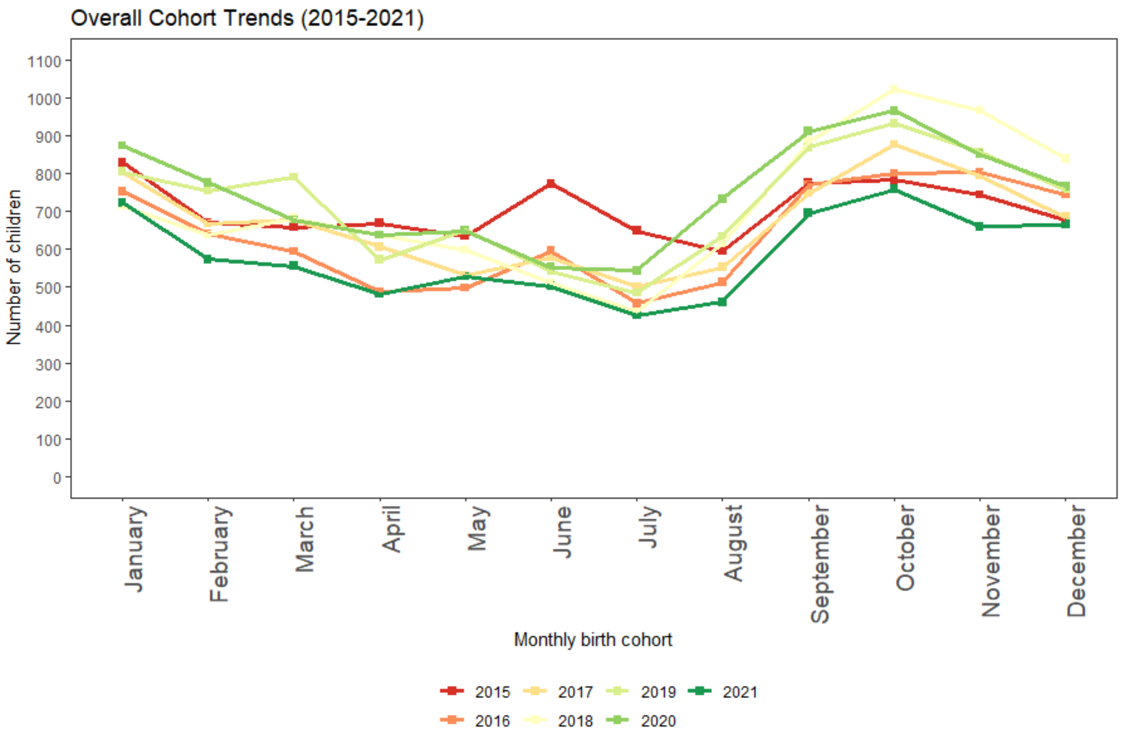

Monthly birth cohort of children included per month, 2015 – 2021 (Basse)

Table S2: Detailed information about the number of eligible children per month from the Basse Health and Demographic Health System in The Gambia, 2015 – 2021

	**Years included in the analysis and eligible children**						
**Months**	**2015**	**2016**	**2017**	**2018**	**2019**	**2020**	**2021**
January	653	557	594	528	597	647	546
February	504	453	476	450	552	575	420
March	475	431	496	502	579	507	402
April	511	364	456	499	444	495	358
May	518	388	391	468	502	468	394
June	550	401	416	386	427	432	389
July	471	329	382	334	390	441	308
August	458	407	419	465	480	548	353
September	611	595	568	682	660	712	527
October	610	623	678	774	742	740	593
November	576	612	616	740	672	658	509
December	500	587	495	644	595	579	544
**Total**	**6437**	**5747**	**5987**	**6472**	**6640**	**6802**	**5343**

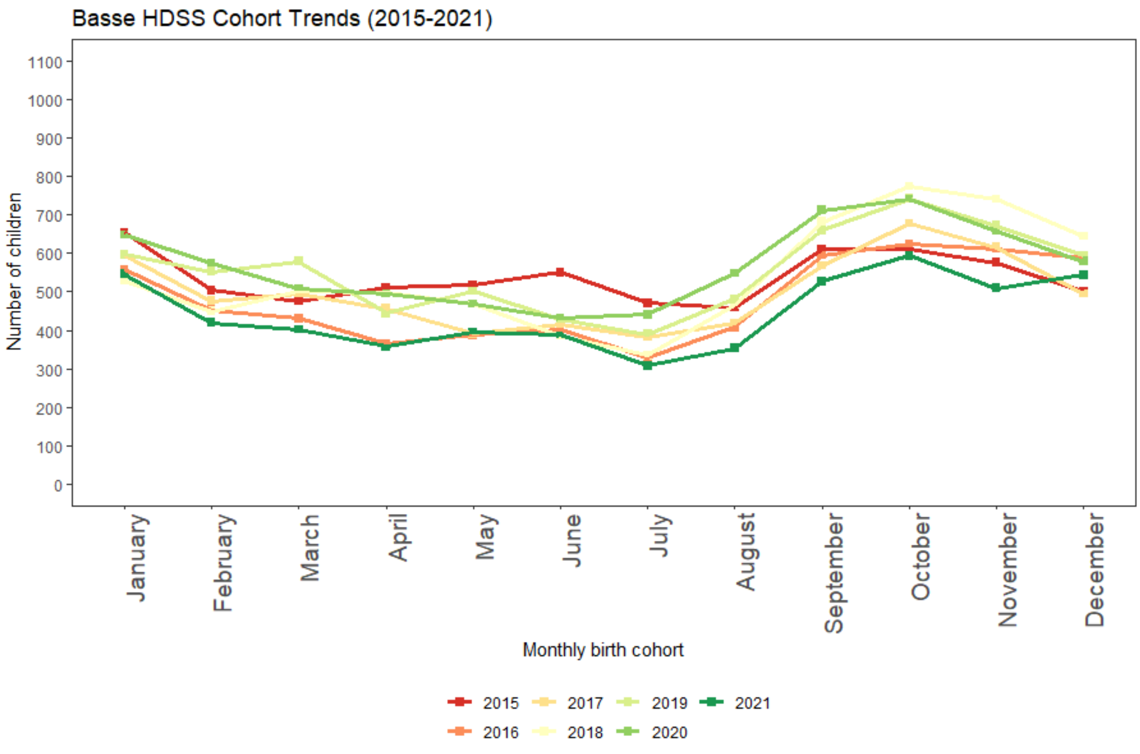

Monthly birth cohort of children included per month, 2015 – 2021 (Farafenni)

Table S3: Detailed information about the number of eligible children per month from the Farafenni Health and Demographic Health System in The Gambia, 2015 – 2021

	**Years included in the analysis and eligible children**						
**Months**	**2015**	**2016**	**2017**	**2018**	**2019**	**2020**	**2021**
January	176	196	209	186	208	226	177
February	166	188	191	187	203	201	153
March	184	163	183	180	211	171	154
April	158	124	151	138	128	142	124
May	117	110	140	130	148	181	134
June	223	195	163	125	114	120	114
July	178	128	120	101	95	103	117
August	138	106	133	150	154	185	108
September	164	174	179	202	209	199	168
October	172	177	199	247	190	225	165
November	168	191	179	226	184	193	151
December	176	157	190	195	162	187	121
**Total**	**2020**	**1909**	**2037**	**2067**	**2006**	**2133**	**1686**

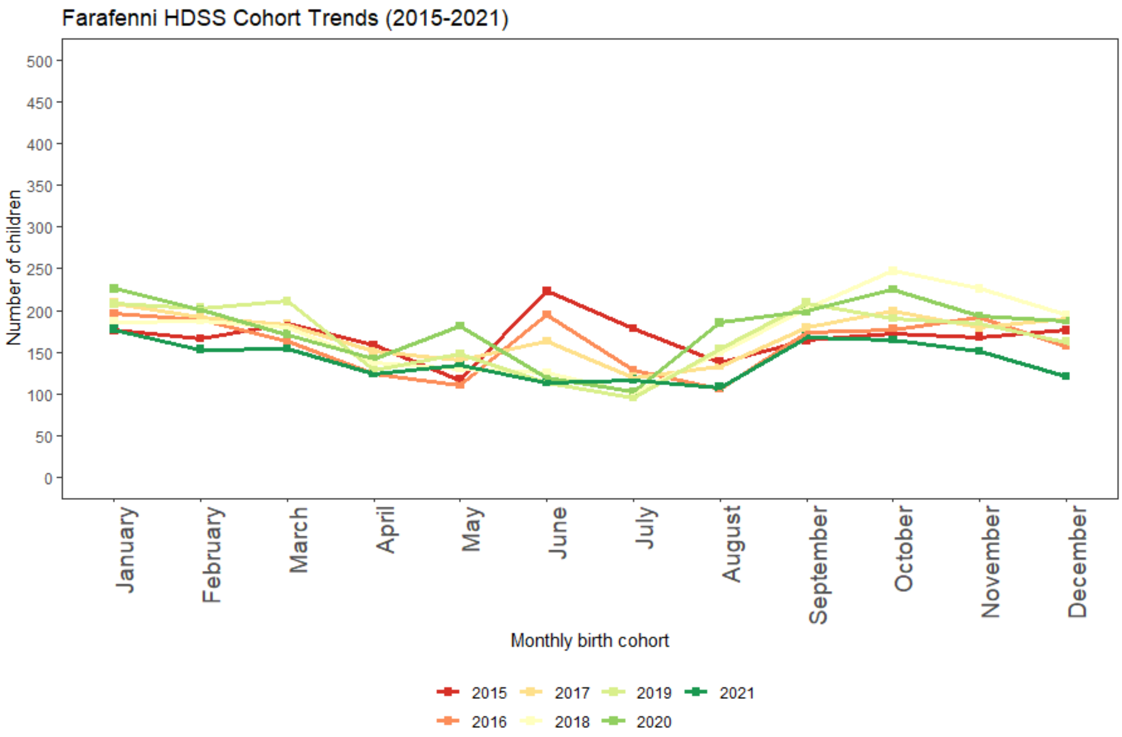

Reasons and numbers of children excluded from the analysis

Table S4: Reasons and numbers of children excluded from the timeliness analysis in Farafenni, Basse, and Overall, January 2015 – December 2021

**Reasons for exclusion**	**Farafenni (N=13,858)** n (%)	**Basse (N=43,428)** n (%)	**Overall (N=57,286)** n (%)
Children with negative vaccination age (HepB0)*	8 (0.06)	321 (0.74)	329 (0.57)
Children with age of hepB0 vaccination more than 150 days**	44 (0.32)	206 (0.47)	250 (0.44)
Children with negative vaccination age (Penta1)*	7 (0.05)	139 (0.32)	146 (0.25)

* These negative age at vaccination (i.e., the difference between the dates of birth and vaccination in days) were likely due to an incorrect date of birth or an incorrect date of vaccination. Because we could not determine which of the two dates was the incorrect one, and because the proportion of affected children was negligible (generally <1%), we decided to exclude them from the timeliness analysis. However, these children were included in the coverage analysis since were certain that they were vaccinated due to the fact that they had a date of vaccination entry.
** We considered that children whose age at HepB) vaccination were >150 days were likely implausible because at that age, they should have received Penta1, Penta2 and Penta3 at 2, 3 and 4 months respectively (60, 90 and 120 days). Although the number of children in this category was negligible (generally <0.5% of the cohort), we considered that such long delays were not likely due to the impact of the pandemic.

Parameter estimates for change in vaccination in Basse HDSS

Table S5: Parameter estimates for the likelihood of change in coverage and the proportion of delayed and early HepB0 and Penta1 vaccinations in the pre-pandemic and pandemic periods in the **Basse HDSS area**

	HepB0			PENTA1		
**COVERAGE**	**Estimate/Odds ratio**	**95% credible interval**		**Estimate/Odds ratio**	**95% credible interval**	
Level change before 1st wave	1.32	0.84	2.05	1.18	0.75	1.84
Level change before 2nd wave	1.09	0.69	1.69	0.89	0.43	1.75
Level change before 3rd wave	1.49	0.86	2.53	1.19	0.47	2.76
Level change AFTER 3rd wave	**2.21**	**1.24**	**3.89**	0.95	0.34	2.37
Pre-pandemic change in slope	0.99	0.99	1.00	0.98	0.97	1.00
Change in slope before 1st wave	1.06	0.93	1.20	1.00	0.86	1.16
Change in slope before 2nd wave	1.06	0.97	1.15	1.05	0.94	1.18
Change in slope before 3rd wave	1.06	0.92	1.22	1.02	0.87	1.19
Change in slope AFTER 3rd wave	0.93	0.78	1.10	1.11	0.92	1.33
$\hat{\sigma}^{-2}$	37.19	20.50	60.76	13.85	5.24	25.93
$\hat{\rho}$	0.50	0.21	0.75	0.83	0.67	0.94
**DELAYED**						
Level change before 1st wave	1.33	0.72	2.46	1.95	0.88	4.55
Level change before 2nd wave	1.41	0.74	2.67	1.12	0.23	4.20
Level change before 3rd wave	1.23	0.58	2.63	0.71	0.09	3.34
Level change AFTER 3rd wave	0.61	0.28	1.36	0.85	0.08	4.56
Pre-pandemic change in slope	**0.97**	**0.97**	**0.98**	1.00	0.98	1.04
Change in slope before 1st wave	1.02	0.85	1.22	0.82	0.61	1.08
Change in slope before 2nd wave	0.97	0.86	1.09	0.92	0.75	1.13
Change in slope before 3rd wave	0.85	0.71	1.01	0.99	0.75	1.31
Change in slope AFTER 3rd wave	1.09	0.87	1.35	0.90	0.64	1.25
$\hat{\sigma}^{-2}$	21.50	9.98	41.54	4.47	1.38	9.17
$\hat{\rho}$	0.54	0.09	0.85	0.82	0.60	0.96
**EARLY**						
Level change before 1st wave				**0.20**	**0.05**	**0.76**
Level change before 2nd wave				0.42	0.15	1.18
Level change before 3rd wave				**0.09**	**0.02**	**0.47**
Level change AFTER 3rd wave				0.45	0.10	1.98
Pre-pandemic change in slope				0.99	0.99	1.00
Change in slope before 1st wave				1.21	0.85	1.73
Change in slope before 2nd wave				0.94	0.75	1.18
Change in slope before 3rd wave				1.43	0.93	2.26
Change in slope AFTER 3rd wave				0.90	0.53	1.51
$\hat{\sigma}^{-2}$				7.73	4.56	12.14
$\hat{\rho}$				0.26	-0.07	0.58

Parameter estimates for change in vaccination in Farafenni HDSS

Table S6: Parameter estimates for the likelihood of change in coverage and the proportion of delayed and early HepB0 and Penta1 vaccinations in the pre-pandemic and pandemic periods in the **Farafenni HDSS area**

	HepB0			PENTA1		
**COVERAGE**	**Estimate/Odds ratio**	**95% credible interval**		**Estimate/Odds ratio**	**95% credible interval**	
Level change before 1st wave	1.01	0.45	2.30	1.14	0.42	3.12
Level change before 2nd wave	2.33	0.88	6.11	3.91	0.92	16.00
Level change before 3rd wave	**2.54**	**1.14**	**11.12**	3.03	0.54	16.04
Level change AFTER 3rd wave	2.29	0.69	7.70	2.97	0.46	17.51
Pre-pandemic change in slope	0.98	0.97	1.00	0.97	0.95	1.13
Change in slope before 1st wave	1.16	0.91	1.49	1.21	0.87	1.68
Change in slope before 2nd wave	1.04	0.87	1.24	1.00	0.78	1.28
Change in slope before 3rd wave	0.87	0.66	1.13	0.97	0.69	1.37
Change in slope AFTER 3rd wave	1.06	0.75	1.48	0.91	0.60	1.39
$\hat{\sigma}^{-2}$	8.25	4.29	13.54	3.54	1.65	6.01
$\hat{\rho}$	0.63	0.40	0.82	0.74	0.56	0.88
**DELAYED**						
Level change before 1st wave	0.97	0.36	2.74	0.87	0.45	1.61
Level change before 2nd wave	2.60	0.84	8.22	1.12	0.72	1.75
Level change before 3rd wave	2.37	0.60	9.57	1.79	0.91	3.50
Level change AFTER 3rd wave	0.38	0.09	1.54	1.38	0.63	3.03
Pre-pandemic change in slope	0.98	0.96	1.00	1.00	0.99	1.00
Change in slope before 1st wave	1.14	0.85	1.55	1.12	0.95	1.35
Change in slope before 2nd wave	0.87	0.71	1.05	1.02	0.93	1.13
Change in slope before 3rd wave	**0.72**	**0.54**	**0.95**	0.88	0.71	1.08
Change in slope AFTER 3rd wave	1.07	0.78	1.46	0.88	0.65	1.19
$\hat{\sigma}^{-2}$	7.99	2.93	18.52	31.75	12.16	74.18
$\hat{\rho}$	0.79	0.43	0.96	-0.14	-0.59	0.36
**EARLY**						
Level change before 1st wave				1.58	0.45	5.59
Level change before 2nd wave				**0.23**	**0.06**	**0.85**
Level change before 3rd wave				1.87	0.43	8.23
Level change AFTER 3rd wave				0.50	0.08	3.01
Pre-pandemic change in slope				1.00	0.99	1.01
Change in slope before 1st wave				0.86	0.59	1.23
Change in slope before 2nd wave				1.31	0.99	1.68
Change in slope before 3rd wave				0.74	0.47	1.12
Change in slope AFTER 3rd wave				1.01	0.53	1.90
$\hat{\sigma}^{-2}$				6.83	3.11	13.19
$\hat{\rho}$				0.50	0.14	0.78

Extra Results: Changes in Penta1 timely vaccination due to the pandemic and counterfactual scenario overall (i.e., Basse and Farafenni HDSS combined)

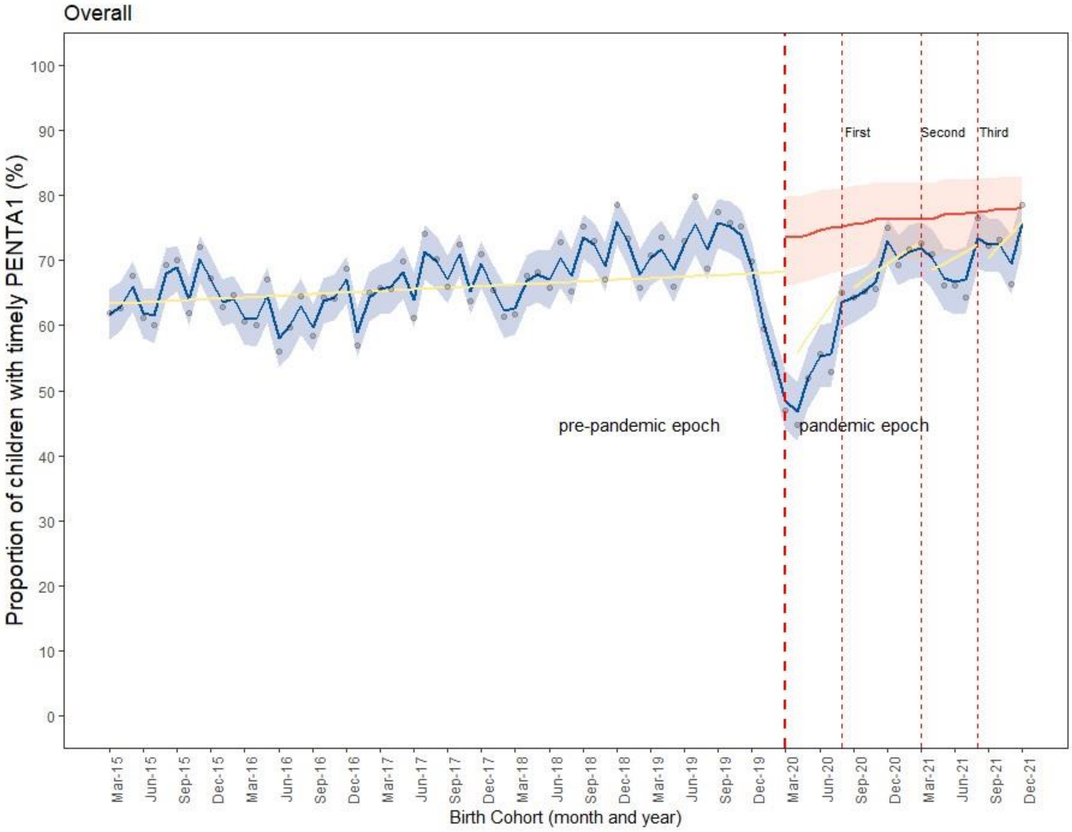

Table S7: Parameter estimates for the likelihood of change in timely Penta1 (Overall)

OVERALL	Odds ratio/Estimate	95% Credible Interval (lower and upper)	
Level change before 1st wave	0.53	0.27	1.03
Level change before 2nd wave	0.83	0.41	2.01
Level change before 3rd wave	0.93	0.41	2.42
Level change AFTER 3rd wave	0.95	0.39	2.61
Pre-pandemic change in slope	1.00	0.99	1.01
Change in slope before 1st wave	1.10	0.92	1.33
Change in slope before 2nd wave	1.05	0.92	1.20
Change in slope before 3rd wave	1.04	0.86	1.27
Change in slope AFTER 3rd wave	1.09	0.86	1.40

*Non of the parameter estimates are significant because the credible intervals include 1.00.

Extra Results: Changes in Penta1 timely vaccination due to the pandemic and counterfactual scenario in Basse HDSS area

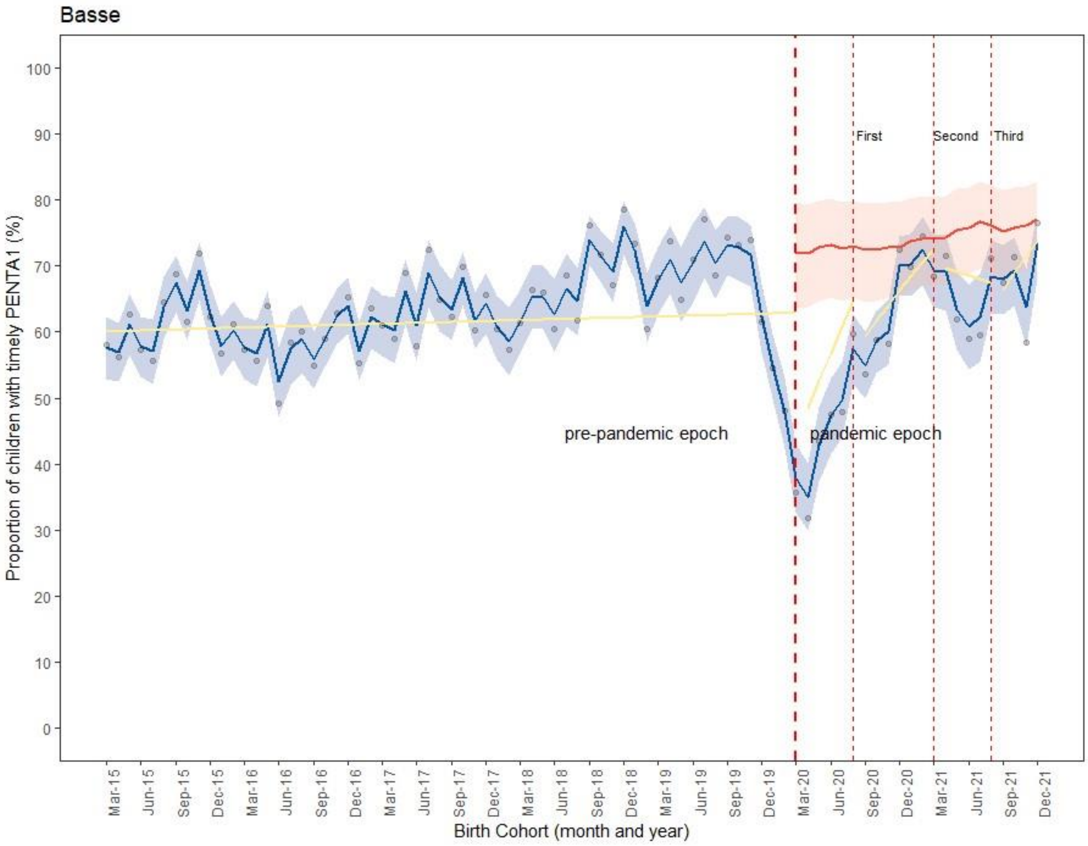

Table S8: : Parameter estimates for the likelihood of change in timely Penta1 (Basse)

Parameters	Odds ratio/Estimate	95% Credible Interval (lower and upper)	
Level change before 1st wave	0.47	0.21	1.00
Level change before 2nd wave	0.77	0.29	2.66
Level change before 3rd wave	1.36	0.44	5.93
Level change AFTER 3rd wave	1.00	0.30	4.96
Pre-pandemic change in slope	1.00	0.98	1.02
Change in slope before 1st wave	1.18	0.94	1.49
Change in slope before 2nd wave	1.10	0.93	1.30
Change in slope before 3rd wave	0.97	0.77	1.23
Change in slope AFTER 3rd wave	1.12	0.84	1.50

*Non of the parameter estimates are significant because the credible intervals include 1.00.

Extra Results: Changes in Penta1 timely vaccination due to the pandemic and counterfactual scenario in **Farafenni HDSS area**

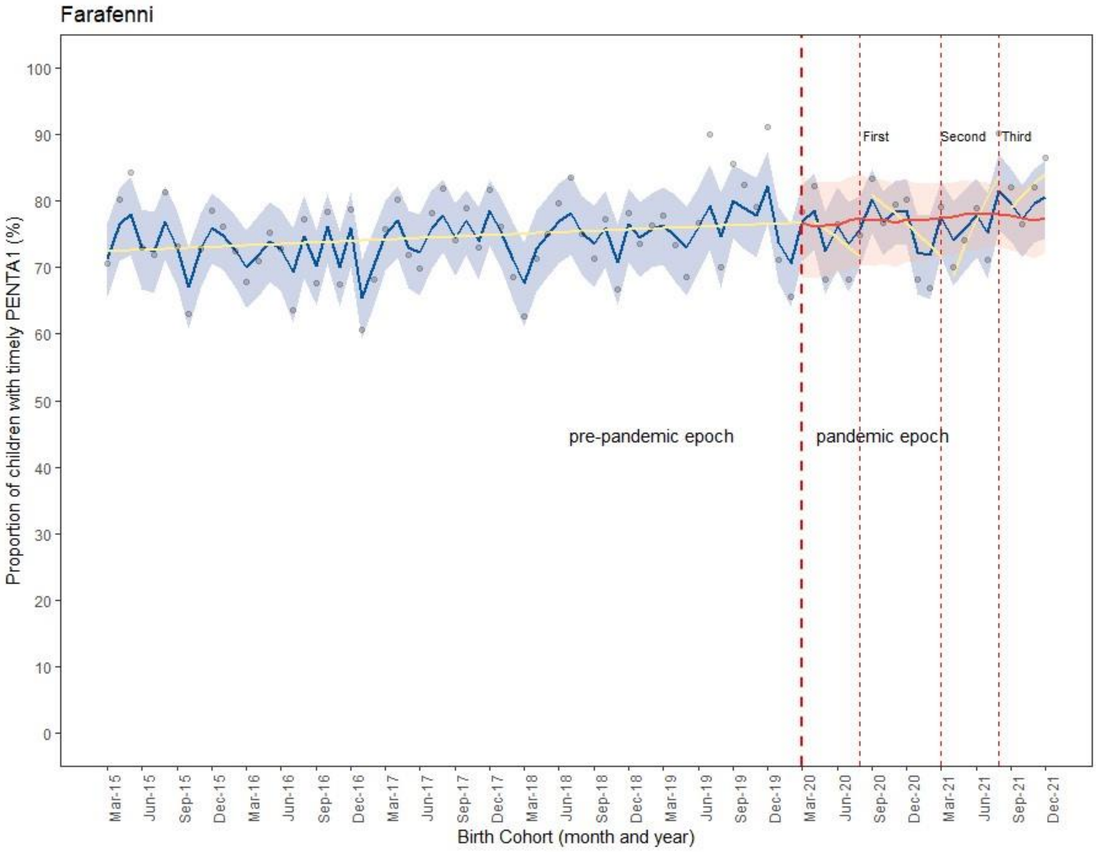

Table S9: Parameter estimates for the likelihood of change in timely Penta1 (Farafenni)

Parameters	Odds ratio/Estimate	95% Credible Interval (lower and upper)	
Level change before 1st wave	1.08	0.53	2.25
Level change before 2nd wave	1.37	0.77	2.42
Level change before 3rd wave	0.51	0.24	1.10
Level change AFTER 3rd wave	0.94	0.38	2.31
Pre-pandemic change in slope	1.01	0.99	1.02
Change in slope before 1st wave	0.93	0.75	1.14
Change in slope before 2nd wave	0.91	0.81	1.03
Change in slope before 3rd wave	1.23	0.98	1.56
Change in slope AFTER 3rd wave	1.12	0.81	1.57

*Non of the parameter estimates are significant because the credible intervals include 1.00.
